# Soluble Beta-Amyloid Precursor Protein Is Related to Disease Progression in Amyotrophic Lateral Sclerosis

**DOI:** 10.1371/journal.pone.0023600

**Published:** 2011-08-15

**Authors:** Petra Steinacker, Lubin Fang, Jens Kuhle, Axel Petzold, Hayrettin Tumani, Albert C. Ludolph, Markus Otto, Johannes Brettschneider

**Affiliations:** 1 Department of Neurology, University of Ulm, Ulm, Germany; 2 Department of Neurology, University of Basel, Basel, Switzerland; 3 Department of Neuroimmunology, UCL Institute of Neurology/National Hospital for Neurology and Neurosurgery, Queen Square, London, United Kingdom; Julius-Maximilians-Universität Würzburg, Germany

## Abstract

**Background:**

Biomarkers of disease progression in amyotrophic lateral sclerosis (ALS) could support the identification of beneficial drugs in clinical trials. We aimed to test whether soluble fragments of beta-amyloid precursor protein (sAPPα and sAPPß) correlated with clinical subtypes of ALS and were of prognostic value.

**Methodology/Principal Findings:**

In a cross-sectional study including patients with ALS (N = 68) with clinical follow-up data over 6 months, Parkinson's disease (PD, N = 20), and age-matched controls (N = 40), cerebrospinal fluid (CSF) levels of sAPPα a, sAPPß and neurofilaments (NfH^SMI35^) were measured by multiplex assay, Progranulin by ELISA. CSF sAPPα and sAPPß levels were lower in ALS with a rapidly-progressive disease course (p = 0.03, and p = 0.02) and with longer disease duration (p = 0.01 and p = 0.01, respectively). CSF NfH^SMI35^ was elevated in ALS compared to PD and controls, with highest concentrations found in patients with rapid disease progression (p<0.01). High CSF NfH^SMI3^ was linked to low CSF sAPPα and sAPPß (p = 0.001, and p = 0.007, respectively). The ratios CSF NfH^SMI35^/CSF sAPPα,-ß were elevated in patients with fast progression of disease (p = 0.002 each). CSF Progranulin decreased with ongoing disease (p = 0.04).

**Conclusions:**

This study provides new CSF candidate markers associated with progression of disease in ALS. The data suggest that a deficiency of cellular neuroprotective mechanisms (decrease of sAPP) is linked to progressive neuro-axonal damage (increase of NfH^SMI35^) and to progression of disease.

## Introduction

Amyotrophic lateral sclerosis (ALS) is the most common form of motor neuron disease characterized by progressive degeneration of spinal and bulbar innervating motor neurons as well as the pyramidal motor neurons [1]. Despite a uniformly fatal outcome, ALS shows a wide range of survival times from a few months to several decades [2]. So far, the pathological determinants of disease progression in ALS remain poorly understood [3]. It is expected that the discovery of sensitive biomarkers of disease progression could be useful for the rapid identification of beneficial drugs in clinical trials, as well as for the prompt exclusion of ineffective ones [4]. Ideally, they could help to shorten clinical trials and limit the need for large placebo-controlled groups [5]. So far, several candidate biomarkers of disease progression have been investigated, though none gained clinical relevance [6], [7]. Cerebrospinal fluid (CSF) is a promising source for biomarkers in ALS since the CSF compartment is in close anatomical contact with the brain interstitial fluid, and could reflect biochemical changes related to the disease [8]. In a previous study on frontotemporal lobar degeneration (FTLD) including a limited number of patients with ALS [9], we analyzed soluble non-amyloidogenic fragments of beta-amyloid precursor protein (APP), sAPPα and sAPPß which were suggested to protect neurons from proteasomal stress [10]. Extending on our previous study, we obtained prospective clinical data on a large cohort of patients with ALS in order to evaluate whether CSF levels of sAPPα and sAPPß would distinguish clinical subgroups of ALS, correlate with disability on clinical scales [11], [12] and would be of prognostic relevance. To evaluate a possible association with neurodegeneration *in vivo* the analyses included an established marker for neuro-axonal damage, the phosphorylated neurofilament heavy chain NfH^SMI35^ [13], [14]. We also analyzed the secreted growth factor Progranulin which was shown to mediate proteolytic cleavage of TAR DNA binding protein of 43 kDa (TDP-43) to evaluate a possible link to the formation of phosphorylated and ubiquitinated aggregates [15].

## Materials and Methods

### Patients and controls

Paired CSF and serum samples were collected of 68 patients with definite sporadic ALS according to revised EL Escorial criteria [16] (Table 1) in a cross-sectional study between January 2008 and September 2009 by the Department of Neurology, University of Ulm (Germany). Clinical follow-up data were collected over a time of 6 months. The disease presented as extremity-onset ALS in 49 patients and as bulbar-onset in 19 in patients. Motor function was quantified clinically using the Medical Research Council grading system (MRCS) with the best score being 60 (full power), testing the patients power on a scale from 0 (no contraction) to 5 (full power) for shoulder abduction, arm flexion, wrist extension, hip flexion, knee extension and ankle dorsiflexion bilaterally [12]. Disability was rated using the revised Amyotrophic Lateral Sclerosis Functional Rating Scale (ALSFRS-R) [11] by two experienced neurologists in our department (CH and AL), each unaware of the biomarker data. At time of lumbar puncture, 61% of patients were treated with Riluzole (50 mg twice a day). Disease progression was evaluated according to the monthly change on the MRCS between baseline and follow-up as previously described (ΔMRCS) [7]. The median was taken as cut-off for statistical purposes. The top 50% were classified as rapidly progressive and the bottom 50% as slowly progressive.

**Table 1:** Demographic data and basic CSF findings of patients included in this study.

	ALS			PD	CTRL	S
		Fast	Slow			
**n (female/male)**	68 (30/38)	34 (16/18)	34 (14/20)	20 (10/10)	40 (20/20)	
	**Median (Range)**					
**Age**	65 (33–84)	67 (33–84)	63 (39–74)	69 (44–86)	62 (21–71)	NS
**Duration of disease [months]**	15 (6–67)	18 (6–67)	16 (6–58)	72 (6–300)		NS*
**MRCS**	56 (33–60)	54 (40–60)	58 (38–60)			
**ΔMRCS**	0.9 (0–9)	2 (0.9–9)	0.2 (0–0.9)			
**ALSFRS**–**R**	40 (6–48)	41 (6–46)	40 (21–48)			
**CSF cell count [cells/µL]**	1 (0–4)	1 (0–4)	1 (0–4)	1 (0–2)	1 (0–4)	NS
**Q_alb_ (x 0.001)**	6.4 (2.3–14.2)	6.6 (2.3–12.4)	5.5 (2.9–14.2)	6.0 (2.8–16.5)	5.4 (2.7–10.6)	NS

*across subgroups of ALS.

ALSFRS-R  =  revised Amyotrophic Lateral Sclerosis Functional Rating Scale, CTRL  =  controls, fast  =  ALS patients with fast progression of disease over follow-up, MRCS  =  Medical Research Council Sumscore, **Δ**MRCS  =  change in MRC score/time**,** NS  =  not significant, PD  =  Parkinson's disease, Q_alb_  =  albumin CSF/serum quotient, slow  =  ALS patients with slow progression of disease over follow-up, S  =  Significance in Kruskal-Wallis One Way Analysis of Variance on Ranks.

The control group consisted of 40 age-matched patients who presented with infrequent episodic tension-type headache [17] and showed no evidence of a structural, hemorrhagic or inflammatory lesion in magnetic resonance imaging (MRI) of the brain. As neurodegenerative disease controls, we included 20 patients with idiopathic Parkinson's disease (PD) [18] (median Hoehn and Yahr) [19] 3, range 1–4) (Table 1). Paired aliquots of 1 ml CSF and serum were stored in polypropylene tubes at −80°C until analysis.

### Ethics statement

Written informed consent was obtained from all patients in accordance with the Declaration of Helsinki, and the study was approved by the ethics committee of the University of Ulm, Germany.

### sAPPα and sAPPß

For determination of sAPPα and sAPPß a multiplex enzyme-linked immunosorbent assay technique based on electrochemoluminescence was applied (Meso Scale Discovery, MSD, Gaithersburg, Maryland, USA) according to the manufacturer's protocols [9]. Analysis plates precoated with 6E10 (Signet Covance, Dedham, MA, USA) as capturing antibody for sAPPα, and ANGU raised against amino acids 591–596 of APP695 [20] as capturing antibody for sAPPß, were blocked and then subjected to 30 µl native CSF or recombinant sAPP dilution series as protein standard. After washing, bound sAPP was detected by P2–1 antibody binding to the APP N-terminal domain [21]. Excess antibody was removed, read buffer was added for 10 min and plates were imaged using a Sector Imager 2400 (MSD, Gaithersburg, USA). Lower limit of detection (LLOD  = 3 SD above the blank signal) for sAPPa was 120 pg/ml, and 52 pg/ml for sAPPß. All samples were measured in duplicates. Detailed information on the analytical performance of this assay and stability of sAPP under storage is provided by a previous study [22].

### NfH^SMI35^

A highly sensitive immunoassay developed in-house for NfH^SMI35^ was used for quantification of NfH in CSF [23]. Briefly, 96-well plates (Multi-Array® plates, Meso Scale Discovery, Gaithersburg, MD) were coated with the capture monoclonal antibody SMI 35R (Covance, Emeryville, CA). Samples were diluted 1∶1 with TBS containing 1% BSA, 0.1% Tween 20 and 50 mM Barbitone (Sigma-Aldrich, Saint Louis, MO). After washing, the secondary polyclonal rabbit anti-NfH antibody (Sigma-Aldrich, Saint Louis, MO) was added to each well. Subsequently the plates were incubated with polyclonal Sulfo-TAG labelled goat anti-rabbit antibody (ruthenylated) (MSD, Gaithersburg, MD), ECL read buffer (MSD) diluted 1∶2 with distilled water and the ECL signals were measured using the MSD Sector Imager 2400 plate reader. A four-parameter weighted logistic fit curve was generated, and sample concentrations extrapolated and analysed using the Discovery Workbench 3.0 software (MSD). In this paper we adhere to a previously proposed nomenclature [24] and indicate the capture antibodies used for NfH phosphoform quantification in superscript NfH^SMI35^. Purified bovine NfH was obtained from US Biological (United States Biological, Swampscott, MA). Standards ranged from 0 to 2500 pg/ml, and the sensitivity of the assay (background plus three standard deviations) is 2.4 pg/ml, with an intra- and inter-assay coefficient of variation of 4.8% and 8.4%. The standards were stored at −20°C. All samples were measured in duplicates.

### Progranulin

Levels of Progranulin in CSF and serum were determined using ELISA according to the instructions as supplied by the manufacturer (Human Progranulin ELISA Kit, AdipoGen Inc., Seoul, Korea). The accuracy of the ELISA was assessed by “Spike-and-recovery” and “linearity-of-dilution” experiments. The serum samples were diluted 1∶600, the CSF samples 1∶12. The sensitivity of the assay (background plus three standard deviations) was 32 pg/ml. All samples were measured in duplicates.

### Statistical analysis

Data analysis was performed using SPSS (Version 17.0 SPSS Inc., Chicago, IL, USA). Because of non-normal data distribution (Kolmogorov-Smirnov test), the medians and interquartile ranges are shown. Differences between two groups were compared using Mann-Whitney Rank Sum Test. To compare raw data of multiple groups, Kruskal-Wallis analysis of variance on ranks was applied, followed in case of significance by Dunn's Method. All correlations were studied using Spearman's rank correlation coefficient. Multiple correlations were corrected using the Bonferroni method. Receiver operating characteristic (ROC) analysis was used to compare the sensitivity and specificity of the ratios CSF NfH^SMI35^/CSF sAPPα and CSF NfH^SMI35^/CSF sAPPßα for identification of ALS patients with a rapid progression of disease. The Youden index was calculated to find the cut-off values which maximize discriminating accuracy of the tests [25]. P-values <0.05 were considered significant.

## Results

### Clinical findings over follow-up

Motor function as measured by the MRCS decreased from a median 56 (range 33–60) points at onset of study to a median 49 (range 7–60) points after 6 months. The median disease progression on the MRCS was 0.92 in this cohort. The ALSFRS-R decreased from a median 40 (range 6–48) points at onset of study to a median of 35 (range 6–46) points after 6 months.

### CSF cytology and albumin

No significant difference of the CSF cytology was observed between patients with ALS and controls (Table 1). A mild blood-CSF barrier dysfunction reflected by an elevated Q_alb_ was observed in 17% of patients with ALS.

### sAPPα and sAPPß

**Figure 1:**
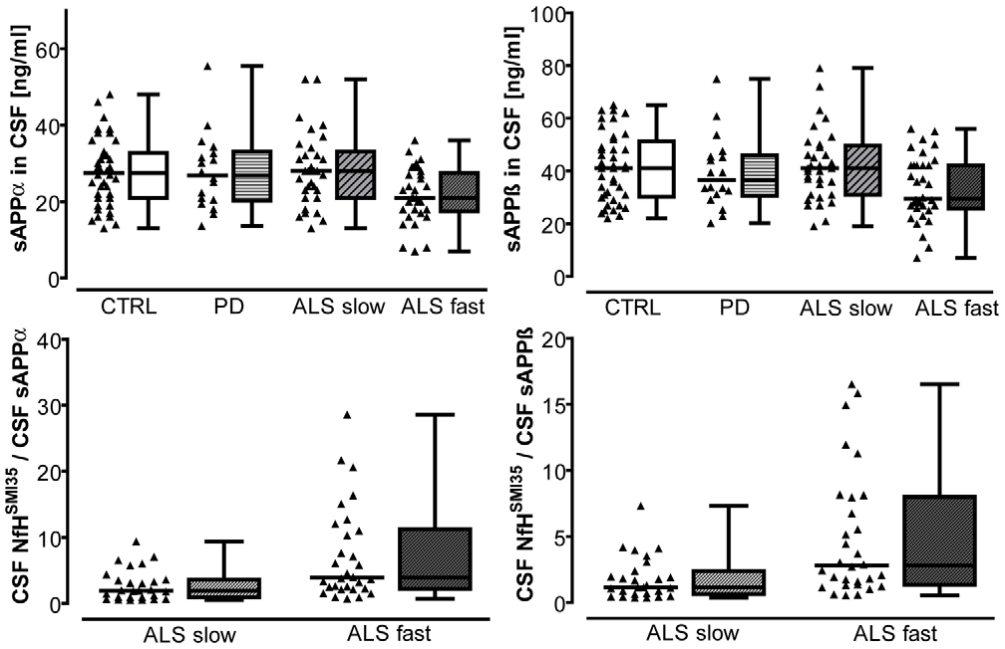
CSF sAPPα/ß in ALS and controls. Box and dot plots show (A) CSF sAPPα and (B) CSF sAPPß in ALS, Parkinson's disease (PD), and controls (CTRL) as well as (C) ratio CSF NfH^SMI35^/CSF sAPPα and (D) ratio CSF NfH^SMI35^/CSF sAPPß (right side). ALS fast  =  patients with rapid progression of disease over follow-up of 6 months, ALS slow  =  patients with slow progression of disease over follow-up. The box represents the 25^th^ to 75^th^ quartile, the whiskers represent the range, and the horizontal line in the box represents the median.

**Figure 2:**
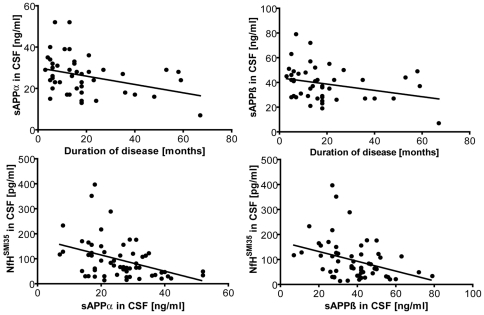
CSF sAPPα/ß in relation to disease progression and NfH. Upper diagrams: Dot plot shows CSF sAPPα and sAPPß in patients with ALS plotted against duration of disease. Straight line represents regression line; correlation was significant (R = −0.39, p = 0.01 for sAPPα and R = −0.37, p = 0.01 for sAPPß). Lower diagrams: Dot plot shows CSF sAPPα and sAPPß in patients with ALS plotted against NfH^SMI35^. Straight line represents regression line; correlation was significant (p = 0.001, R = −0.42 for sAPPα, and p = 0.007, R = −0.35 for sAPPß).

Both CSF sAPPα and CSF sAPPß concentrations were decreased in a subgroup of ALS patients with a rapid-progressive course of disease (p = 0.03, and p = 0.02, Figure 1). For all ALS patients combined, no significant difference of CSF sAPPα and CSF sAPPß concentrations to controls and PD was observed (p = 0.45, and p = 0.35). Both CSF sAPPα and sAPPß decreased as motor function was progressively lost (change of MRC score/time) (R = −0.3, p = 0.04 for sAPPα, R = −0.3, p = 0.01 for sAPPß). Both sAPPα and sAPPß were decreased in patients with extremity onset of disease as compared to patients with bulbar onset (p = 0.04, p = 0.02). No difference of CSF sAPPα or sAPPß between ALS patients with and without Riluzole was observed (p = 0.54, p = 0.61). CSF sAPPα was lower as disease duration increased (R = −0.39, p = 0.01, Figure 2). Similarly, sAPPß was lower as disease duration increased (R = −0.37, p = 0.01, Figure 2). There was a strong correlation of CSF sAPPα with CSF sAPPß levels (R = 0.89, p<0.001). For neither sAPPα nor sAPPß a correlation between CSF and serum levels was observed (p = 0.57, p = 0.15, respectively). No correlation of sAPPα or sAPPß with clinical scores (MRCS or ALSFRS-R) at time of lumbar puncture was observed (data not shown). Neither CSF sAPPα nor sAPPß correlated with blood-CSF barrier function as measured by Q_alb_ (p = 0.1, and p = 0.09). Furthermore, no correlation of CSF sAPPα or CSF sAPPß with age of patients was detectable (p = 0.6, and p = 0.2).

### NfH^SMI35^ and ratio NfH^SMI35^ to sAPPα, sAPPß

**Figure 3:**
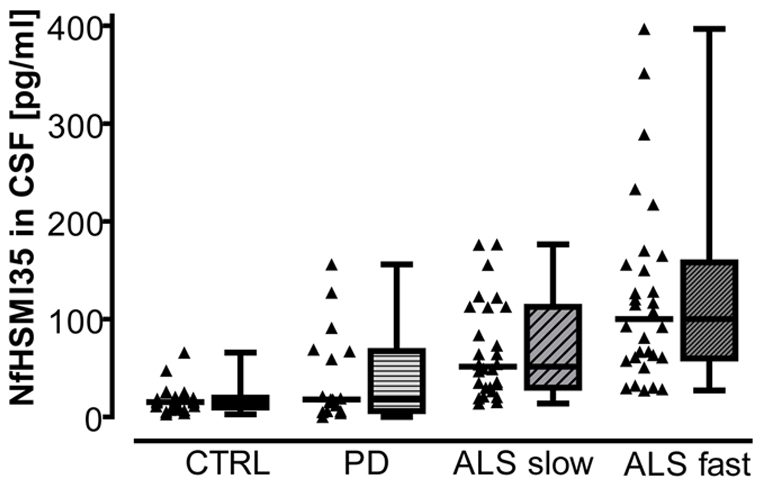
CSF NfH in ALS and controls. Box and dot plots show CSF NfH^SMI35^ in ALS, Parkinson's disease (PD), and controls (CTRL). ALS fast  =  patients with rapid progression of disease over follow-up of 6 months, ALS slow  =  patients with slow progression of disease over follow-up. The box represents the 25^th^ to 75^th^ quartile, the whiskers represent the range, and the horizontal line in the box represents the median. Difference between the groups was significant (p<0.001, Kruskal-Wallis Analysis of Variance on Ranks), with post-hoc analysis (Dunn's method) showing patients with ALS to have significantly higher CSF concentrations as compared to patients with PD and controls (p<0.05 each).

CSF NfH^SMI35^ was higher in patients with ALS as compared to PD and controls (p<0.05 each, Figure 3). Furthermore, CSF NfH^SMI3^ was elevated in the subgroup of patients with a rapid progression of disease (p = 0.01, Table 2). There was no correlation of CSF NfH^SMI35^ levels with either age (p = 0.7), disease duration (p = 0.09) or blood-CSF barrier function (p = 0.52). No difference of CSF NfH^SMI3^ between patients with bulbar and extremity-onset (p = 0.62) or with and without riluzole was detectable (p = 0.42). CSF NfH^SMI3^ decreased with increasing CSF sAPPα and sAPPß concentrations (p = 0.001, R = −0.42 for sAPPα, and p = 0.007, R = −0.35 for sAPPß, Figure 2). This correlation was also observed in the subgroup of patients with a rapid progression of disease (p = 0.01, R = −0.45 for sAPPα, and p = 0.04, R = −0.38 for sAPPß).

**Table 2:** CSF and Serum sAPPα, sAPPß, NfH^SMI35^, and Progranulin (PRGN) in patients with ALS, Parkinson's disease (PD), and controls (CTRL).

	ALS			PD	CTRL	S
		Fast	Slow			
	Median (Range)					
**CSF sAPPα** **[ng/ml]**	24 (7–52)	21 (7–36)	28 (13–52)	27 (14–55)	28 (13–48)	p = 0.03*
**Ser sAPPα** **[ng/ml]**	285 (128–493)	310 (235–493)	278 (128–426)	294 (188–597)	334 (159–547)	NS
**CSF sAPPß** **[ng/ml]**	37 (7–79)	29 (7–56)	41 (19–79)	37 (20–75)	41 (22–65)	p = 0.02*
**Ser sAPPß** **[ng/ml]**	44 (15–310)	48 (29–310)	44 (15–63)	48 (19–82)	50 (24–121)	NS
**CSF NfH^SMI35^** **[pg/ml]**	67 (14–397)	108 (27–397)	52 (14–177)	18 (4–156)	15 (3–66)	p<0.001‡
**CSF PRGN** **[ng/ml]**	3.9 (2.2–8.2)	4.2 (2.2–7.4)	3.5 (2.6–8.2)	4.1 (2.6–4.5)	4.3 (2.3–7.0)	NS
**Ser PRGN** **[ng/ml]**	105 (52–269)	123 (63–235)	98 (52–269)	110 (65–196)	109 (79–185)	NS

‡ Comparison across all groups, Kruskal-Wallis Analysis of Variance on Ranks.

*Comparison of ALS fast vs. ALS slow, Mann-Whitney Rank Sum Test.

Fast  =  ALS patients with fast progression of disease over follow-up, slow  =  ALS patients with slow progression of disease over follow-up, S  =  statistical significance.

We observed a difference of the ratio CSF NfH^SMI35^/CSF sAPPα between ALS patients with fast progression of disease and a slower progression of disease during the prospective follow-up period (p = 0.002, Figure 1). Similarly, we observed a difference of the ratio CSF NfH^SMI35^/CSF sAPPß between ALS patients with fast progression of disease and a slower progression of disease over follow-up (p = 0.002).

### ROC-Analysis

Using ROC analysis, we determined a ratio CSF NfH^SMI35^/CSF sAPPα of 2.1 (AUROCC 0.75, sensitivity 0.84, specificity 0.60, Youden index 0.43) and a ratio CSF NfH^SMI35^/CSF sAPPß of 4.3 (AUROCC 0.74, sensitivity 0.83, specificity 0.59, Youden index 0.41) as optimal cut-off to differentiate patients with a fast progression of disease from patients with a slower progression of disease.

For NfH^SMI35^, ROC analysis yielded a cut-off of 55.8 pg/ml (AUROCC 0.7, sensitivity 0.8, specificity 0.57, Youden index 0.37), for CSF sAPPα a cut-off of 27.5 ng/ml (AUROCC 0.66, sensitivity 0.53, specificity 0.74, Youden index 0.25), and for sAPPß a cut-off of 29.5 ng/ml (AUROCC 0.67, sensitivity 0.82, specificity 0.5, Youden index 0.32) to differentiate patients with a fast progression of disease from patients with a slower progression of disease.

### Progranulin

We observed no difference of either CSF or serum Progranulin concentrations between patients with ALS, PD and controls (p = 0.8, and p = 0.4). Similarly, there was no difference of CSF Progranulin between subgroups of ALS patients with fast and slow disease progression (p = 0.07). Likewise there was no difference of CSF Progranulin between patients with bulbar or extremity-onset (p = 0.67) or treatment with Riluzole (p = 0.17). We observed no correlation of CSF Progranulin with age of patients (p = 0.12). We also found no correlation of CSF Progranulin with blood-CSF barrier function as measured by Q_alb_ (p = 0.9). We observed an inverse correlation of CSF Progranulin levels with duration of disease (p = 0.04, R = −0.32). No correlation of serum levels with duration of disease was detectable (p = 0.78).

## Discussion

We observed CSF levels of sAPPα and sAPPß to be altered in subgroups of ALS: They were a) decreased in patients with a rapid-progressive course of disease over follow-up (Figure 1) and b) in patients with extremity-onset of disease. Furthermore, both CSF sAPPα and sAPPß were found to decrease with ongoing disease (Figure 2). In accordance with previous observations, CSF sAPPα and sAPPß concentrations were strongly correlated [9], [22]. With regard to the specificity of the assay used to determine sAPPα/ß, a recent study revealed no significant cross-reactivity of antibodies specific for sAPPα or sAPPß with other CSF proteins [22].

Cleavage of APP by the α-and ß-secretase pathway results in the production of the sAPPα or sAPPß fragment [26]. As sAPPα and sAPPß are mainly produced by neurons,[27] decrease of CSF sAPP was suggested to reflect loss of functional neurons in neurodegenerative disease [28]. Consequently, decreasing CSF sAPPα and sAPPß with ongoing disease as detected in our study could reflect progressing neuronal loss or dysfunction in ALS. The mechanisms underlying low CSF sAPP in patients with extremity-onset are so far unclear. No difference regarding the extent and distribution of protein aggregates in the lower motor neuron columns with regard to site of disease onset was observed [29]. We speculate that lower CSF sAPP levels are related to the larger number of neurons lost in extremity onset compared to bulbar onset disease. Secreted APPα was suggested to have potent neuroprotective capacities: It binds to a specific receptor linked to cyclic-GMP (cGMP) production and activation of cGMP-dependent protein kinase (PKG), promoting activation of the nuclear transcription factor NF-κB; these effects of sAPPα are believed to mediate its neuron-survival-promoting properties [30]. Furthermore, sAPPα was found to protect neurons from proteasomal stress [31] by inhibiting the stress-triggered pro-apoptotic c-Jun N-terminal kinase (JNK)-signaling pathway [10]. Impaired proteasomal function is a major hallmark in the pathophysiology of neurodegenerative diseases, and it may explain the increased ubiquitination and presence of proteinaceous aggregates such as Nf in ALS [32]. Consequently, low CSF sAPPα concentrations observed in patients with a rapid-progressive course of disease could mirror a deficiency of neuronal mechanisms involved in protein degradation and protection against misfolded or damaged proteins in ALS. With decreased sAPP levels one may expect more NfH aggregates to develop.

We found an inverse correlation of both sAPPα and sAPPß levels with NfH^SMI35^ in the CSF (Figure 2). In a previous study, we observed high concentrations of NfH^SMI35^ in ALS to mirror extensive axonal damage and indicate a rapid progression of disease [7]. In accordance with our previous study, we observed NfH^SMI35^ to be elevated in the CSF of patients with ALS as compared to all other groups (Figure 3), with highest concentrations found in patients with a rapid-progressive course of disease over follow-up. The inverse correlation of CSF sAPPα and sAPPß with CSF NfH^SMI35^ which was prominent in patients with a rapid progression of disease suggests that low CSF concentrations of sAPP in ALS are linked to extensive neuro-axonal damage. Our data support the relevance of sAPPα and -ß as neuroprotective agents and provides *in vivo* evidence that a deficiency of cellular mechanisms protective against the formation of proteinaceous aggregates could be a determinant of disease progression in ALS. As both high CSF NfH^SMI35^ and low CSF sAPPα and -ß were associated with a rapid deterioration of motor function over follow-up, we determined a combined analysis of those markers. The ratios CSF NfH^SMI35^/CSF sAPPα and CSF NfH^SMI35^/CSF sAPPß were superior to NfH^SMI35^ and sAPPα,ß alone to delineate patients with a fast progression of disease and are therefore promising candidate biomarkers of disease progression for clinical trials of ALS. In analogy to what has been proposed for combining CSF tau and abeta levels in Alzheimer's disease we propose combining CSF NfH and sAPP in ALS. However, there was an overlap between the groups and the number of patients included was comparatively low, so the cut-offs determined here using ROC-analysis will have to be validated on a large cohort of patients with ALS. We propose this to be done in an unbiased, staged multicenter, validation strategy.

Though no significant difference of CSF or serum Progranulin between patients with ALS and controls was detectable, we observed a tendency of CSF Progranulin to decrease with ongoing disease. Progranulin mediates proteolytic cleavage of TDP-43 to generate ∼35 and ∼25 kDa species [15]. Suppression of Progranulin expression was shown to lead to caspase-dependent accumulation of TDP-43 fragments [15]. Decreasing CSF Progranulin levels with ongoing disease as observed in our study could mirror deficient cleavage of TDP-43 and could contribute to the formation of proteinaceous aggregates in ALS.

Our data provides indirect biomarker *in vivo* evidence that a deficiency of neuroprotective mechanisms involved in protein degradation and cleavage is linked to progressive neuro-axonal damage in ALS. On a clinical level, this study provides new CSF candidate markers associated with progression of disease in ALS.
